# Liaison psychiatry services in Scotland, 2022: online survey of NHS health boards

**DOI:** 10.1192/bjb.2023.68

**Published:** 2024-08

**Authors:** Prakash Shankar, Murray Smith

**Affiliations:** 1 Forth Valley Royal Hospital, Larbert, UK; 2 Aberdeen Royal Infirmary, Aberdeen, UK

**Keywords:** Liaison psychiatry, service development, Scotland, staffing, psychiatric nursing

## Abstract

**Aims and method:**

Liaison psychiatry services have seen significant developments the UK. Regular surveys of liaison psychiatry in England have contributed to this, but it has not attracted the same interest in Scotland, with only a mention and no commitments in the Scottish Government's Mental Health Strategy. There have been no comprehensive surveys in Scotland and this study was an attempt to explore provisioning of services. A questionnaire was sent to liaison psychiatry services in the mainland Scottish National Health Service (NHS) health boards.

**Results:**

Responses obtained from all 11 boards revealed considerable variation in service provisioning. Services provided through acute rather than mental health directorates seem significantly better resourced.

**Clinical implications:**

Liaison psychiatry services can improve care for patients but require adequate resources to do so. There are limited quality standards for Scottish liaison services, unlike other devolved nations, leading to variation in provision. This survey will assist in designing quality standards for liaison psychiatry in Scotland.

The Scottish Government's Mental Health Strategy 2017–2027 describes liaison psychiatry as the psychiatric subspecialty that provides advice, assessment, treatment and training regarding patients in emergency departments, general hospital wards and out-patient services.^1^ These services can benefit patients with comorbid physical and mental health problems by reducing lengths of stay, reducing readmissions and investigations, and improving care of ‘medically unexplained symptoms’ and chronic health conditions. They are needed when use of statutory mental health legislation is required, which poses particular problems in general hospitals.^2^ The case for cost-effectiveness of liaison psychiatry services has been made by various authors.^3,4^

There has been no previous comprehensive survey of liaison psychiatry services in Scotland, although it has been self-evident that services are variable across the country. National standards such as those published by the Royal College of Psychiatrists (RCPsych), some in collaboration with other medical Royal Colleges,^5,6,7,8^ have been instrumental in defining services. These have not been adopted or implemented consistently in Scotland, unlike in other parts of the UK. In England, there has been a regular survey of liaison services conducted for several years, facilitated by Public Health England and the RCPsych. The RCPsych in Scotland Liaison Faculty was keen to replicate the process to improve and develop services based on local priorities. This might lead to better service structures, such as managed clinical networks or similar structures for equity of service provisioning, and achieving similar accreditation standards consistent with other devolved nations of the UK.

Planning and dissemination of information for this first survey of liaison psychiatry services in Scotland was undertaken in the first quarter of 2022. At that time there were 33 full-time equivalent (FTE) consultants in liaison psychiatry in the 11 mainland National Health Service (NHS) health boards, providing services to 29 hospitals. The inequitable distribution of liaison services was stark and inconsistent with acceptable standards specified in RCPsych guidance.^9^ Furthermore, the island boards of Orkney, Shetland and Western Isles had no specific liaison psychiatry service and depended on *ad hoc* cover from general adult psychiatric services. This will clearly have an impact on the consistency of care of these patients during presentations to acute hospitals^6^ and affect services' ability to reduce the burden of long-term conditions.^10^

NHS England's commissioning of sequential national surveys of liaison psychiatry services in England (LPSEs), especially LPSE-2,^11^ led to incremental growth of liaison psychiatry services in England. The strategy led to emergence of the Five Year Forward View, which aspired to establishing Core 24 liaison services everywhere in England by 2021.^12^ Initiatives such as the Achieving Better Access to Mental Health Services programme^13^ were able to further the parity of esteem agenda. These building blocks of liaison psychiatry services perhaps helped quicken the adaptive changes necessitated by the COVID-19 pandemic.^14^ The Welsh Government was able to follow similar strategic initiatives, which required all district general hospitals in Wales to have a liaison psychiatry team in place by March 2017.^15^

Scotland's 2017–2027 Mental Health Strategy^1^ had proposed increasing the workforce to give access to dedicated mental health professionals for all emergency departments, general practices, police station custody suites and prisons with an additional investment of £35 million for 800 additional mental health workers in these key settings. Despite the strategic directions, there was a dearth of policy directives or clear funding streams to facilitate expansion of liaison services to provide Core 24 services as described, for example, in the standards of the RCPsych's Psychiatric Liaison Accreditation Network (PLAN).^9^ In England this was specified in the Five Year Forward View^12^ with specific funding and a series of surveys to check progress. Even though clinical needs and clinical standards are similar in Scotland and England, no such centrally mandated and funded growth has happened in Scotland. This remains a missed opportunity to set standards and replicate progress in other areas of the UK. The Scottish Government is currently consulting to update self-harm guidelines, and expansion of liaison services to provide Core 24 standards will become inevitable. Therefore, a comprehensive survey of all the liaison psychiatry services in acute hospitals in Scotland became necessary.

## Method

The RCPsych in Scotland Liaison Faculty undertook mapping of the current provisioning of liaison psychiatry services in Scotland. For the purposes of consistency we followed a methodology similar to that used by the LPSEs and the National Service Group for Liaison Psychiatry, Public Health Wales.^17^ This mapping exercise to explore service provisioning did not require approval from an ethics committee.

The structured questionnaire (supplementary material, available at https://dx.doi.org/10.1192/bjb.2023.68) was based on LPSE-5 and Psychiatric Liaison Accreditation Network (PLAN) standards.^9^ This facilitated information gathering on the level of service provision, the make-up of liaison psychiatry teams by discipline, the type of service provided, the clinical areas and hospitals covered, the hours of working and service governance arrangements. The first draft of the questionnaire was piloted to test for suitability and for ease of data collection at a regional super-specialty centre. Subsequent iterations were based on feedback to improve the quality of data collection. Locality-based lead clinicians for liaison psychiatry services were identified in each health board in Scotland. The questionnaire, which included questions on team structure, referrals and assessment facilities, teaching and training, and recording of outcomes, was disseminated electronically to this link person in each health board in the fourth quarter of 2021 and followed up by email reminders in first quarter of 2022. The data were collected and analysed using Microsoft Excel.

## Results

At least one response was obtained from each of the 11 mainland health boards. There are 30 hospitals with emergency departments in Scotland. In each of these boards there is some liaison psychiatry provisioning to acute hospitals with emergency departments, illustrated in Table 1. This configuration might be altered given the Scottish Government's strategic direction for greater integration with local authorities to form health and social care partnerships.

**Table 1:** Hospitals emergency departments and liaison psychiatry service provision

Health board	Hospitals with emergency departments	Is there a liaison psychiatry team?	Emergency department covered by liaison psychiatry team?	Wards covered by liaison psychiatry team?
Ayrshire & Arran	University Hospital Ayr	Yes (based off-site)	Yes	Yes
	University Hospital Crosshouse	Yes	Yes	Yes
Borders	Borders General Hospital	Yes	Yes	Yes
Dumfries & Galloway	Dumfries & Galloway Royal Infirmary	Yes	No	Yes
	Galloway Community Hospital	No		
Fife	Victoria Hospital	Yes	Yes	Yes
Forth Valley	Forth Valley Royal Hospital	Yes	Yes	Yes
Grampian	Aberdeen Royal Infirmary	Yes	Yes	Yes
	Dr Gray's Hospital	Yes	Yes	Yes
	Royal Aberdeen Children's Hospital	No		
Greater Glasgow and Clyde	Glasgow Royal Infirmary	Yes	Yes	Yes
	Inverclyde Royal Hospital	Yes	Yes	Yes
	Royal Alexandra Hospital	Yes	Yes	Yes
	Royal Hospital for Children Glasgow	CAMHS		
	Queen Elizabeth University Hospital	Yes	Yes	Yes
Highland	Belford Hospital	No		
	Caithness General Hospital	No		
	Lorn & Islands Hospital	No		
	Raigmore Hospital	Yes	Yes	Yes
Lanarkshire	University Hospital Hairmyres	Yes	Yes	Yes
	University Hospital Monklands	Yes	Yes	Yes
	University Hospital Wishaw	Yes	Yes	Yes
Lothian	Royal Hospital for Sick Children Edinburgh	CAMHS		
	Royal Infirmary of Edinburgh	Yes	Yes	Yes
	St John's Hospital	Yes	No	Yes
Orkney	Balfour Hospital	No		
Tayside	Ninewells Hospital	Yes	Yes	Yes
	Perth Royal Infirmary	No		
Shetland	Gilbert Bain Hospital	No		
Western Isles	Western Isles Hospital	No		

CAMHS, child and adolescent mental health services.

### Adult mental health services

Most adult teams have age boundaries – usually working age (aged 18–64 years) and older adults (65 and over), although some working age teams cover presentations of self-harm in older adults. We did not find specific liaison psychiatry services in NHS Orkney, Shetland, or Western Isles, each of which has one main emergency department, and the provisioning is dependent on wider general adult services.

### Child and adolescent mental health services (CAMHS)

There are isolated child and adolescent mental health liaison teams (e.g. the Paediatric Psychology and Liaison Service, PPALS, in Edinburgh), with cover provided in other areas either by in-reach from local CAMHS or some cover by adult liaison services.

### Service structure

There is variation across the country, with 13 working age liaison psychiatry teams, 11 older adult teams and 5 teams that cover both working age and older adults. Teams reported working across 29 general hospital sites (which do not all have emergency departments, for example the Western General Hospital in Edinburgh) and a few teams provide some community hospital cover. The largest liaison team is based at the Royal Infirmary in Edinburgh and the smallest in Raigmore in Inverness.

Scotland has 20 hospitals with liaison psychiatry teams that have an emergency department, although 3 of those emergency departments are covered by an alternative service and the liaison team only covers in-patient wards; 17 emergency departments have input from the working age liaison team, although one of those does not have specific cover from an older adult team. Eight emergency departments in Scotland do not have any specific liaison psychiatry cover.

### Place of safety

Thirteen liaison psychiatry teams cover assessments under section 297 of the Mental Health (Care and treatment) (Scotland) Act 2003 (the police's power to remove a person to a designated place of safety). In other centres, this is either covered by a duty psychiatry team or the board's designated place of safety is an alternative site.

### Staffing

Table 2 summarises the staffing levels for the liaison services by discipline. Several teams provide cover at multiple hospital sites and there are different structures and remits of services, so direct comparison is not possible. There were 33 FTE liaison psychiatry consultants (24.5 for working age adult services, 9.4 for all adults and 8.3 for older adult services). There were approximately 108 liaison psychiatry nurses, with 8 teams having one or fewer FTE consultants. Most of the consultants were based in the central belt of the country (covered by hospitals in NHS Greater Glasgow and Clyde, Lanarkshire and Lothian), with most other board areas having one or two main hospitals with emergency departments, with one main liaison psychiatry team. There is only a small amount of integrated psychology resource, and this is only available in a small number of teams. The numbers Table 2 reflect approximate totals across all sites and all patient age ranges, and in some centres, nursing and other resources are shared with other services and are not a specific liaison psychiatry resource. Table 3 provides an approximate illustration of the average numbers of liaison psychiatrists per 1000 in-patient beds they cover per health board.

**Table 2:** Staffing levels for the liaison psychiatry services by discipline

Board	Emergency department	Team base	Consultants (FTE)	Other medical	Nurses (FTE)	Psychology (FTE)	Allied health professionals (FTE)
Ayrshire & Arran	University Hospital Ayr	Off-site	4	Rotational junior doctor	21.26	0.1	0
	University Hospital Crosshouse	On-site					
Borders	Borders General Hospital	On-site	1	0	4	0	0
Dumfries & Galloway	Dumfries & Galloway Royal Infirmary	Off-site	0.1	0	6.3	0	0
	Galloway Community Hospital	n.a.					
Fife	Victoria Hospital	Off-site	0.8	0	4	0	0
Forth Valley	Forth Valley Royal Hospital	On-site	1.7	Rotational junior doctor	8.1	1	1
Grampian	Aberdeen Royal Infirmary	On-site	1.3	Rotational junior doctor	14.1	0.4	0
	Dr Gray's Hospital	On-site					
	Royal Aberdeen Children's Hospital	n.a.					
Greater Glasgow & Clyde	Glasgow Royal Infirmary	On-site	7.8	Specialty doctor Rotational junior doctor	30	3.5	0
	Inverclyde Royal Hospital	Off-site					
	Royal Alexandra Hospital	On-site					
	Royal Hospital for Children Glasgow	Off-site					
	Queen Elizabeth University Hospital	On-site					
Highland	Belford Hospital	n.a.	0	Sessional input	4.6	0	0
	Caithness General Hospital	n.a.					
	Lorn & Islands Hospital	n.a.					
	Raigmore Hospital	On-site					
Lanarkshire	University Hospital Hairmyres	On-site	3.5	Part-time junior doctor	5.8	0.5	0
	University Hospital Monklands	On-site					
	University Hospital Wishaw	On-site					
Lothian	Royal Hospital for Sick Children Edinburgh	Off-site	11	Rotational junior doctor	12	1	0
	Royal Infirmary of Edinburgh	On-site					
	St John's Hospital	On-site					
Orkney	Balfour Hospital	n.a.					
Shetland	Gilbert Bain Hospital	n.a.					
Tayside	Ninewells Hospital	On-site	1.6	Rotational junior doctor	6.7	1	0
	Perth Royal Infirmary	n.a.					
Western Isles	Western Isles Hospital	n.a.					

FTE, full-time equivalent; n.a., not applicable.

**Table 3:** Average number of liaison psychiatrists per 1000 in-patient beds

Health board	Total liaison psychiatry consultants (FTE)	Approximate liaison psychiatry consultants per 1000 in-patient beds
Ayrshire & Arran	4	3
Borders	1	3
Dumfries & Galloway	0.1	0.3
Fife	0.8	1.1
Forth Valley	1.7	1.8
Grampian	1.35	1
Greater Glasgow and Clyde	7.8	1.9
Highland	0	0
Lanarkshire	3.5	2.5
Lothian	11	9
Orkney	0	0
Shetland	0	0
Tayside	1.6	1.6
Western Isles	0	0

FTE, full-time equivalent.

### Funding

The 2017–2027 Mental Health Strategy suggests funding for liaison psychiatry services through acute services.^1^ This is only the case for two teams in Lothian, with funding through various medical specialties, and for an older adult nurse in Grampian. All other liaison psychiatry teams are funded through mental health services.

### Hours of operation

The medical staff in the liaison teams in the survey work 09.00–17.00 h Monday to Friday, with any medical cover for liaison out of hours being covered by on-call teams. Most of the teams operate the same or similar hours, with two teams operating extended hours into evenings. Only one team operates over 24 h. A small number of teams have a limited weekend service for ‘self-harm’ assessments. Most services indicated that outside of core working hours, there is no specific liaison cover and responsibility would fall to a duty or on-call team. All teams have some form of handover system for interfacing with out-of-hours teams.

### Other related resources

In many acute hospitals there are resources that may have a related but separate remit to liaison psychiatry, and close working and links with these teams or individuals can benefit patients. Two teams have an integrated alcohol specialist nurse service. One team said there is no specific resource for addiction. Most centres note a separate drug and alcohol service, which falls under the responsibility of either acute medical or addiction services. Eight teams report availability of an intellectual disability nurse. One team has access to a dedicated pharmacist, and most other centres reported access to either acute care pharmacists or mental health pharmacists as needed.

No services have specialist health psychology or neuropsychology support integrated into their teams, although a few describe pockets of this attached to specific medical specialties, but access to and interface with these resources is variable. These types of service often have an overlapping remit and better integration with liaison psychiatry services may provide a better service to patients.

### Accommodation

Most teams are based in the main hospital in which they work but may have to travel to peripheral sites. Four teams are not based on-site and are based in a local community resource centre or psychiatric hospital. Several teams report insufficient or no specific office space and the need for hot-desking. Some teams report that moves due to the COVID-19 pandemic have contributed to this.

For clinical assessments of patients, PLAN standards require emergency departments to have an appropriate assessment area, with fixed furniture and two outward-opening doors that are not lockable from the inside.^9^ Seven teams reported that emergency departments adequately meet these requirements, but several others reported there being no such space. Similarly, ward-based assessments are often reportedly conducted at the bedside because of lack of suitable spaces. For liaison psychiatry teams that offer an out-patient service, the PLAN suggests either adequate office space or that these clinics are conducted remotely.

### Referrals and response times

All teams have referral criteria available for referrers consistent with RCPsych (CR183) standards.^2^ Most teams use either an online or email referral system, although two teams exclusively take referrals by telephone or in person. All report that they can respond to emergency referrals within the same day, along with urgent referrals. Most teams usually see routine referrals within 3 days, except for some specialist assessments. Most teams have an audit system, or system in development, to monitor this.

### Out-patient resources

Specialist psychiatric liaison out-patient resources appear very variable. Six liaison services in the organisations with acute hospitals and emergency departments have out-patient resources. These include brief intervention (including one perinatal brief intervention) clinics, complex liaison clinics for patients with, for example, comorbid physical and mental disorders, or functional disorders, and delirium follow-up.

### Teaching and training

All teams offer teaching and training to medical and nursing students, with many teams offering clinical attachments. All offer teaching and training to local emergency departments and other medical teams. Some teams contribute to local junior doctor induction programmes. Most team members report access to adequate training opportunities, reflective practice sessions and supervision.

### Quality, audit and governance

The responses on outcome measures revealed that three teams have documents recording key performance indicators and one has a document in development. The other teams reported no specific document. Eight teams measure patient outcomes and one has a measure in development.

Five teams record specific patient feedback regularly, three record this occasionally and three have feedback forms in development. Two older adult teams describe difficulty in obtaining patient feedback owing to the nature of presenting symptoms in the patient populations they serve.

## Discussion

### Findings in context

The summary of results from this survey of liaison psychiatry services in Scotland reveals wide variation in service provision across Scotland. There is evidence to suggest that in some health boards there is no specific liaison psychiatry provisioning and the boards rely on stretched community mental health teams and duty psychiatrist cover. Several liaison psychiatry teams have only a small provision of staff, often with only one (or fewer) FTE consultants in liaison psychiatry, which leaves these services unable to provide Core 24 services. Many areas, outside of core working hours, rely on in-reach from duty teams, which is not supported by the evidence base for general hospital liaison, and ‘crisis teams’, which are distinct teams that have different functions and skills.

The results also reveal flourishing liaison psychiatry service provisioning in the central belt of the country, with local populations benefiting significantly. There are pockets of good practice, with specialised addiction, child and adolescent, perinatal, intellectual disability and older adult services. In some areas, acute services funded liaison psychiatry provision through specialist areas such as organ transplantation, gender dysphoria and self-harm services. These services seem well resourced, with secure funding streams from the acute services.

There are few psychology and allied health professionals, such as social workers, occupational therapists or physiotherapists, integrated into liaison teams, so patients in Scotland do not have the opportunity to receive comprehensive therapeutic inputs, for example brief psychological interventions in medical and surgical wards, along with consultation, training and reflective practice for teams.^18^

### Strengths and limitations

The strength of the study is the comprehensive response rate, which enabled accurate characterisation of current service configurations. The informal network of liaison psychiatrists in Scotland clarified the collated information where needed. Limitations of the survey included an inability to identify the nature of the organic development of liaison services locally to identify the factors that led to inequitable distribution of liaison service provisioning. The results of this survey will be influenced by the quality of information provided by the respondents. The possibility of significant changes in service configuration cannot be ruled out owing to the changing ecosystem of service provisioning.

### Implications

Despite these limitations, the results of this study have profound implications. Health Boards in Scotland have autonomously established services according to local needs and requirements. Scotland is diverse in its geography and demography, with a mix of rural and urban centres, and the size of geographical areas covered by local organisations varies.

To reduce the variation and provide consistency in service provisioning, the survey offers an opportunity for services to aspire to well-established benchmarks for liaison psychiatry with minimum recommended staffing numbers depending on the size of the hospital and type of service, as elucidated in Table 4.^2^

**Table 4:** High-level summary of differences between care models^a^

	Core	Core 24	Enhanced 24	Comprehensive
Approximate number of beds	500	500	500	2000
Consultants	2	2	4	5
Other medical	0.6	2	2	2
Nurses	2 Band 7 6 Band 6	6 Band 7 7 Band 6	3 Band 7 7 Band 6	2 Band 8b 17 Band 6 10 Band 5
Other therapists	0	4	2	16
Team manager Band 7	1	1	1	3
Clinical service manager Band 8	0.2	0.2–0.4	0.2–0.4	1
Administration Bands 2, 3 and 4	2.6	2	2	12
Business support (Band 5)	0	1	1	1
Total full-time equivalents	14.4	25.2–25.4	22.2–24.4	69
Hours of service	09.00–17.00	24/7	24/7	24/7
Patient age, years	16+	16+	16+	16+
Older adults	Yes	Yes	Yes	Yes
Drug and alcohol service	No	Yes	Yes	Yes
Out-patient	No	No	Yes	Yes
Specialties	No	No	No	Yes
Approximate cost	£0.7 million	£1.1 million	£1.4 million	£4.5 million

a. Numbers of staff are shown as full-time equivalents.

Source: Baugh & Talwar (2022).^9^

Additionally, recently updated quality standards from the PLAN can help build a case for robust liaison psychiatry services.^9^ This will be a challenging proposition, as evaluation of services is dependent on the alignment of incentives driving such an evaluation. The regulatory role of the Care Quality Commission (CQC) influences commissioning bodies to invest in liaison psychiatry services to make the provision of these services more attractive. Responsibility for health remains a devolved matter and is legislated by the Scottish Parliament, with no distinct delineation of provider–purchaser split. Therefore, a force field analysis in Scotland will have different driving and restraining forces. Different health boards might thus invest in developing services in line with their local vision and perceived requirements. But the persistent variation in level of provision between areas remains impossible to justify. The 2017–2027 Scottish Mental Health Strategy missed the opportunity to set standards that would have led to an expansion of services similar to that which occurred with liaison services in England. This inevitably left the field open for organisations to make their own decisions, which unfortunately did not result in appropriate expansion. The number of liaison services funded through acute streams rather than mental health is the same as it was when the strategy was launched. Therefore, the next logical stage from this initial survey should be efforts to quantify unmet needs of the local population based on referral rates and waiting times based on socioeconomic disparities in order to develop liaison services. There are putative efforts to develop standards such as those published by PLAN for liaison psychiatry services in centres of excellence in Scotland and this needs replication across other areas. The opportunity cost of developing new standards will have to be weighed against implementing the standards that have evolved in England.^8^ Most fully developed liaison services are those funded through acute hospitals, which also have high levels of specialist funding, and this is not a coincidence.

There is an opportunity to build on the iterative process adopted by the LPSEs in England so that the mapping exercise over a period will establish aspirational benchmarks. Such comparisons will emphasise the need for funding arrangements to develop liaison psychiatry services in Scotland. There will also be an opportunity to reduce challenges related to artificial boundaries of age (such ageless liaison services), neurodevelopmental conditions and intellectual abilities.^19^

Liaison psychiatry services are highly sought after by trainees to develop confidence in managing crisis and complex comorbidities, including medically unexplained illness.^20^ Therefore, expansion of liaison services will attract trainees to areas that struggle with such placements. Much of the expansion in Lothian has been funded by specialist medical services, including liver transplant, renal transplant, nephrology, diabetes, infectious diseases, haemophilia and trauma. This is welcome but piecemeal (as similar inputs for other specialties, such as gastrointestinal, cardiology, respiratory services, is lacking) and is no basis for a systematic upscaling unless underpinned by strategic initiatives.

The wide geographical variation in provision of liaison psychiatry services in Scotland is impossible to justify. It is therefore vital to build on the impetus from the first survey of its kind in Scotland with a view to setting and adhering to quality standards and conducting further surveys to improve the health outcomes of the Scottish population. The RCPsych remains dedicated to having liaison psychiatry service provisioning for every acute hospital^21^ and this is not yet the case in Scotland.

## Supporting information

Shankar and Smith supplementary material Shankar and Smith supplementary material

## Data Availability

The data that support the findings of this study are available from the corresponding author, M.S., upon reasonable request.

## About the authors

**Prakash Shankar** is a consultant liaison psychiatrist in the Liaison Psychiatry Mental Health Acute Assessment Team at Forth Valley Royal Hospital, Larbert, UK. **Murray Smith** is a consultant liaison psychiatrist in the Department of Psychological Medicine at Aberdeen Royal Infirmary, Aberdeen, UK.
