# Supplementary material for: Liaison psychiatry services in Scotland, 2022: online survey of NHS health boards
Source: BJPsych Bull. 2024 Aug;48(4):226–33. doi: 10.1192/bjb.2023.68 (PMC11543307; doi:10.1192/bjb.2023.68)
Supplement: Shankar and Smith supplementary material [file S2056469423000682sup001.pdf]

1. **Health Board** ______________________________

2. Please fill in box to describe Hospitals and Liaison Services in your Health Board:

Hospital	Liaison Team Base	ED Y/N	Approx. No of beds	Does Liaison Team Cover ED?	Does Liaison Cover S.297?	Liaison funded by Mental Health or Acute services?

3. Liaison Service Staffing

**In Hours**	Dedicated Team – Wards?	Dedicated Team – ED?	Hours of operation	Based on Site?	Consultant WTE	Nursing Grades/WTE	Other Medical	Trainees	Psychology	AHPs	Other (i.e. peer worker)
Working Age											
Older Adult											
CAMHS											

Comments:

**Out of Hours**	Dedicated Team – Wards?	Dedicated Team – ED?	Hours of operation	Based on Site?	Consultant WTE	Nursing WTE	Other Medical	Trainees	Psychology	AHPs	Other (i.e. peer worker)
Working Age											
Older Adult											
CAMHS											

Comments:

4. Additional staffing

**Drug & Alcohol Team**	Attached	Separate	None	Comments

**LD Nurse/equivalent**	Attached	Separate	None	Comments

**Pharmacist**	Attached	Separate	None	Comments

**Other services** i.e. Health Psychology	Attached	Separate	None	Comments

5. Additional Staffing cont.

Are there regular reviews of staffing requirements (i.e. annual)?

Are there adequate handover arrangements to out of hour's/on call teams? Please detail:

Does the team have adequate office space (please comment on i.e. hot-desking, IT, not on site)? :

Does the team have regular meetings?

	Referrals	Clinical	Business
Frequency			

6. Liaison Team Referrals

	In Hours	Out of Hours
Are contact details for assessment advice easily accessible (i.e. on intranet)?		
Are referrals Criteria available for acute staff (i.e. on intranet)?		

Is there a standard referral form?	Online	Email	Paper	Other

What are the team's standard Response times	Emergency	Urgent	Routine	Are these/how are these monitored?

Does the team have access to adequate assessment facilities?<br><br>(i.e. room with fixed furniture/2 doors etc.)	ED	Wards	Outpatient

Does the Liaison team have an outpatient resource? Please describe remit:

7. Teaching & Training

Does the Liaison Team offer teaching and training to medical and nursing students?

Does the Liaison Team offer teaching and training to acute hospital colleagues?

Do Liaison Team members have access to adequate training/professional development opportunities?

Do all Liaison Team members have access to clinical supervision?

Do all Liaison Team Members have access to reflective practice sessions?

8. Quality/audit/governance

Is there a written document describing key performance indicators?

If yes, do you believe the KPI document represents a good measure of outcomes from your services?

Are clinical outcomes measured?

Is patient feedback gathered?

Are these data used to review/develop the service?

Comments:
